# Daptomycin Failure for Treatment of Pulmonary Septic Emboli in Native Tricuspid and Mitral Valve Methicillin-Resistant *Staphylococcus aureus* Endocarditis

**DOI:** 10.1155/2013/653582

**Published:** 2013-11-25

**Authors:** Hadeel Zainah, Marcus Zervos, Wassim Stephane, Sara Chamas Alhelo, Ghattas Alkhoury, Allison Weinmann

**Affiliations:** Infectious Diseases Department, Henry Ford Hospital, 2799 W. Grand Boulevard, CFP-304, Detroit, MI 48202, USA

## Abstract

Daptomycin has been used with success for the treatment of right-sided methicillin-resistant *Staphylococcus aureus* (MRSA) endocarditis. However, its efficacy has not been completely assessed for the treatment of MRSA endocarditis when it is associated with pulmonary septic emboli. Hereby, we present a case of MRSA mitral and tricuspid native valve endocarditis with pulmonary septic emboli, which was treated with daptomycin as a sole agent, resulting in worsening pulmonary infiltrates and treatment failure.

## 1. Introduction

Methicillin-resistant *Staphylococcus aureus* (MRSA) endocarditis has high morbidity and mortality especially when septic emboli are present [1]. Different treatment regimens have been endorsed for the treatment of endocarditis [1]. Daptomycin has been proven to be efficacious for the treatment of right-sided MRSA endocarditis [2], but there is no evidence to support its efficacy when septic emboli are present. Daptomycin is usually inactivated in the presence of surfactant.

## 2. Case Presentation

A 24-year-old female with active intravenous drug use (IDU) presented to the hospital with fever, shortness of breath, chills, generalized weakness, productive cough, back pain, and bilateral flank pain. She had a recent history of *Escherichia coli* pyelonephritis two weeks prior to admission, for which she was being treated with Ciprofloxacin 500 mg orally twice a day. The patient was a current smoker and used intravenous heroin daily in addition to smoking marijuana. 

**Figure 1:**
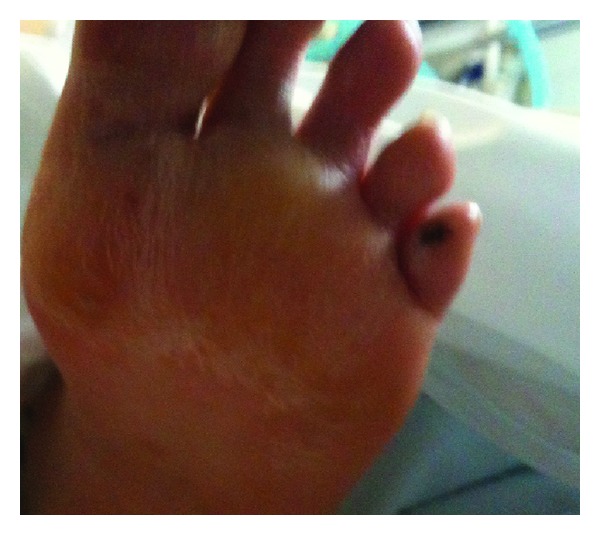
Skin lesion on the left foot.

Physical exam revealed thin-built chronically ill-appearing lady. Oral temperature was 36.5°C, heart rate was 87/min, blood pressure was 117/59, respiratory rate was 17/min, and oxygen saturation was 99% on 2 L of oxygen. She had left shoulder tenderness and limited range of motion, tenderness on the cervical and lumbar spine, weakness in the lower extremities, and bilateral flank pain. No bruits were detected on cardiac exam. Lungs were clear bilaterally. Lesions were noted on palms and soles (Figure 1). The rest of the examination was normal.

Laboratory studies showed white blood cell count: 16.5 K/*μ*L [3.8–10.6], hemoglobin: 8.8 mg/dL [12–15], platelets: 260 K/*μ*L [150–450], and creatinine: 1.78 mg/dL [<1.03].

Blood cultures showed methicillin-resistant *Staphylococcus aureus* (6 days of sustained bacteremia). 

**Figure 2:**
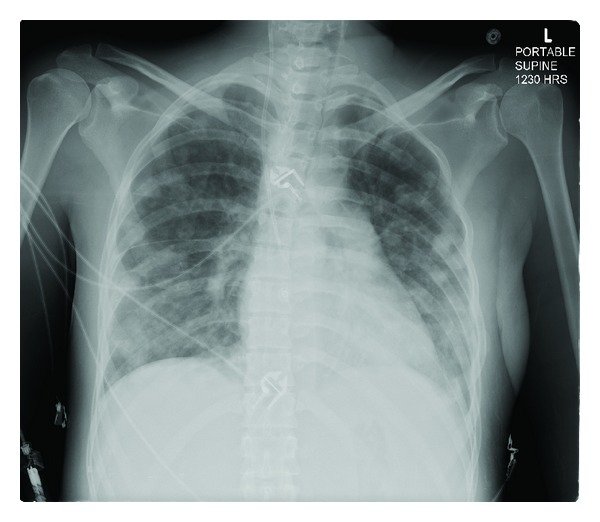
Chest X-ray showing pulmonary septic emboli.

**Figure 3:**
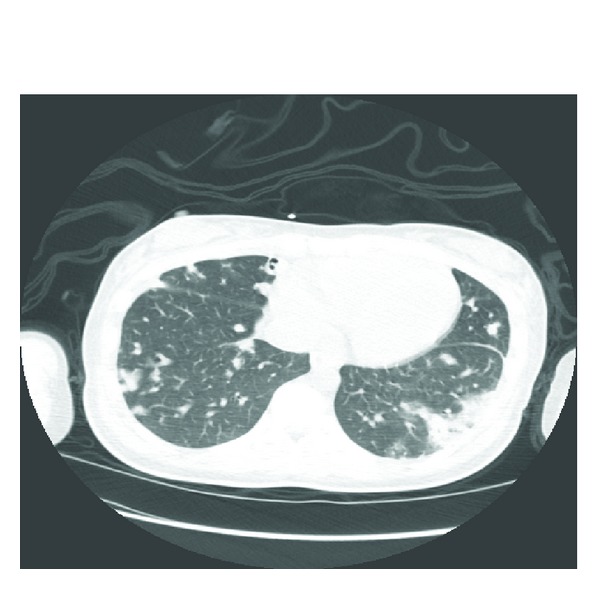
Chest CT scan showing pulmonary nodules.

Transesophageal echocardiogram showed mitral and tricuspid valve vegetation and severe tricuspid regurgitation. Chest X-ray (Figure 2) and tomography (Figure 3) showed multiple lung nodules compatible with septic emboli. Magnetic resonance imaging (MRI) of the brain showed cerebral and cerebellar emboli. Spinal MRI was negative for spinal infection. Ultrasound of the left shoulder was negative for joint effusion. 

**Figure 4:**
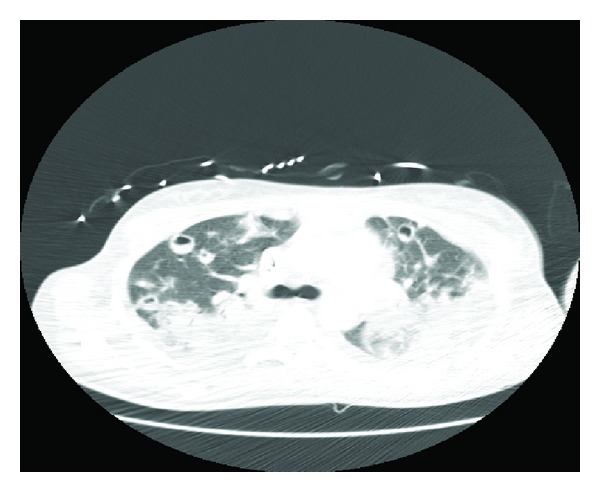
Chest tomography showing development of pulmonary infiltrates.

**Figure 5:**
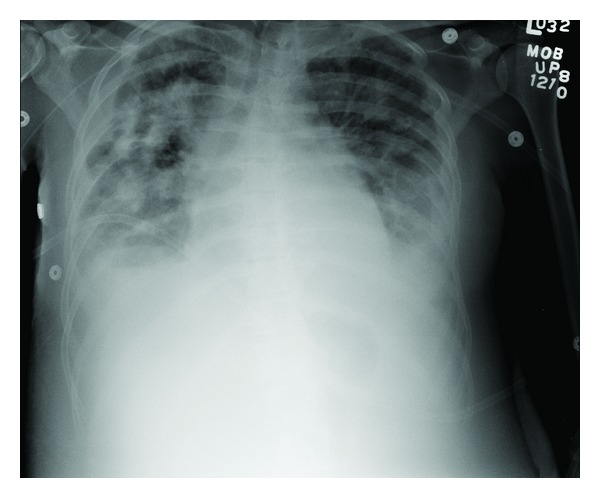
Chest X-ray showing progression of diffuse pulmonary infiltrates.

The patient was started on daptomycin (6 mg/kg IV daily) since vancomycin was avoided due to the presence of acute renal injury. On day 4, chest tomography showed progression in the number and size of cavitary lesions (Figure 4). She remained hemodynamically stable with overall improved respiratory status including decreasing oxygen requirements of 2 liters and was transferred to a general medical floor on day number 7; daptomycin was continued as the sole antimicrobial. On day 11, the patient became febrile and chest X-ray showed diffuse airspace disease and cavitary lesions (Figure 5). Daptomycin was switched to ceftaroline (600 mg IV twice daily). On day 17, repeat tomography showed decrease in size of multiple cavitary and noncavitary nodules with patchy airspace disease. The patient was discharged to rehabilitation center; there was resolution of infection at 6 weeks.

## 3. Discussion

MRSA infections could be acquired either in the healthcare setting or in the community [3]. MRSA endocarditis is common in intravenous drug users [4]; this association was first recognized in 1950. The incidence is higher in younger patients when associated with IDU [5]. MRSA endocarditis has less-favorable outcome and higher rate of complications in intravenous drug users [6]. Larger vegetations carry higher mortality and poor prognosis [6].

Septic pulmonary emboli are usually seen in right-sided endocarditis and to a lesser degree in deep tissue infections as described by Lin et al. [7]. 

Our patient had community-acquired MRSA bacteremia and subsequently both right-and left-sided endocarditis due to IDU. The disease was complicated with pulmonary septic emboli as a result.

Daptomycin has been shown to be effective in the treatment of MRSA endocarditis both in endocarditis experimental models [8] and in clinical studies [9]. Furthermore, it was found to be noninferior to vancomycin for MRSA endocarditis treatment [2]. It has been suggested that it can be used in patients with endocarditis with septic emboli; however, it has not been completely evaluated for this purpose. It is known to be inactivated by alveolar surfactant, which eliminates its use in pneumonia; that has been shown in vitro as the first organ-specific inhibition of an antibiotic [10]. There are limited data on the use of daptomycin for therapy of endocarditis with septic pulmonary emboli. In the Fowler Jr. et al. study, there were only 10 patients with septic emboli in the daptomycin arm, but they were not analyzed separately in comparison with vancomycin [2]. Thus, daptomycin should be used with caution for the treatment of MRSA endocarditis with pulmonary septic emboli.

On the other hand, vancomycin's efficacy in the treatment of pulmonary disease has been established; it is considered the drug of choice for MRSA pneumonia [11, 12]. Ceftaroline has also been proven effective in the treatment of community-acquired pneumonia [13–15]. Additionally, it was used in the treatment of MRSA bacteremia and even endocarditis as revealed in a recent case series of 31 patients with MRSA bacteremia including 9 patients with endocarditis with a high success rate [16].

Our patient was initially started on daptomycin instead of standard therapy with vancomycin due to acute renal injury, the septic emboli were initially small, and daptomycin was a plausible option. Daptomycin was continued despite the initial worsening of the pulmonary infiltrates due to stable clinical status and oxygen requirements. The patient subsequently became febrile and worsening pulmonary infiltrates developed despite daptomycin treatment. Daptomycin was changed to ceftaroline; the patient improved and was discharged after completion of therapy and resolution of infection.

This case report emphasizes caution when daptomycin is used for the treatment of endocarditis complicated with pulmonary septic emboli as efficacy is diminished in the lungs due to surfactant inactivation. Respiratory status of patients should be carefully monitored and, if there is clinical deterioration, consideration should be given to a change in antimicrobial cover. More information is needed for the use of this agent in this setting.
